# ‘Magic cosmetic fillers’: Appearance-enhancement effects on self–face recognition

**DOI:** 10.1371/journal.pone.0305580

**Published:** 2024-06-13

**Authors:** Valentina Cazzato, Charlotte Ellis, Stergios Makris

**Affiliations:** 1 School of Psychology, Faculty of Health, Liverpool John Moores University, Liverpool, United Kingdom; 2 Department of Cognitive Sciences, Psychology, Education and Cultural Studies, University of Messina, Messina, Messina, Italy; 3 Department of Psychology, Edge Hill University, Ormskirk, United Kingdom; 4 Arts and Wellbeing Research Centre, Edge Hill University, Ormskirk, United Kingdom; Universita degli Studi di Udine, ITALY

## Abstract

People naturally exhibit a self-serving bias which can be observed in their tendency to judge their own physical attractiveness more favourably than that of others. Despite this positive self-perception, minimally invasive cosmetic injectable procedures for facial rejuvenation and enhancement are becoming increasingly common. It remains unclear, however, whether recognizing an altered version of one’s own face, enhanced cosmetically, correlates with a positive view of cosmetic surgery and excessive preoccupations about physical characteristics perceived as defects (body dysmorphic concerns). In this study, 30 healthy female participants, aged 18–24 years (Mage = 21.1 years, SD = 1.6), engaged in a face recognition task during which their faces were digitally morphed with that of gender-matched unfamiliar women who had undergone cosmetic enhancements, specifically lip and cheek fillers. The duration of exposure to these modified faces varied with short (500 msec) and long (2000 msec) viewing periods. Participants were asked to identify whether the digital morphs represented themselves or the other woman. Self-reports regarding acceptance of cosmetic surgery and dysmorphic concerns were collected. Participants PSE indicated a tendency towards self-bias under short presentation times, shifting towards the other as presentation times lengthened. Interestingly, this effect was associated with greater acceptance of cosmetic surgery and higher body dysmorphic concerns. This study underscores the importance of understanding how perceptions of others’ physical appearances can influence self-recognition and attitudes towards cosmetic surgery, which may have both positive and potentially harmful implications.

## Introduction

Every morning, we glance at the mirror to assess our appearance. Whenever we observe our reflection, we inherently recognize it as our own. The significance of the self-face lies in its uniqueness, playing a special role in shaping our identity and contributing to our sense of self. Unlike information related to faces of others, our face is a singular stimulus, unique to everyone and not shared with others [1]. This is demonstrated by the fact that one responds faster to one’s own face than to the faces of others, a phenomenon known as the ’self-face advantage’ (see [2] for a review). Interestingly, people generally hold a self-enhancement bias whereby they perceive their own face as more attractive than it might be [3–5]. The underlying mechanism of this phenomenon is not fully understood, but one possibility could be the identification with attractive others through blurring of self–other boundaries [6]. Traditionally, self-face recognition with another face has been long explored in studies where participants’ faces were morphed with attractive others. For instance, a seminal study by Epley and Whitchurch [5] found that participants were more likely to recognise a more attractive version of their face as their own, suggesting that this could be a form of self-enhancement, driven by implicit and automatic psychological mechanisms. This bias also correlated with measures of self-worth, hinting at a top-down effect where positive self-associations lead to positive self-distortions. Similarly, previous studies have found an egocentric bias in self–other facial morphing tasks, during which a more attractive relative to a less attractive face is presented [6]. For example, a recent study by Panagiotopoulou et al. [6] showed that the attractiveness of others can cause positive self-distortions, with individuals more likely to identify with more attractive others, potentially due to blurring self-other boundaries. In contrast, individuals with psychiatric conditions like schizophrenia [7], anorexia nervosa (AN, [8]) and borderline personality disorder [9], show an ‘alter-centric bias (i.e., judging self–other morphs to look more like “other” than “self”) where they identify more with others than themselves. Individuals suffering from AN have been found to exhibit significantly greater difficulties than controls in identifying their own face, which in turn was associated to nutritional state [8]. The exact mechanism behind this bias in face recognition is unclear, but it may involve a blurring of bodily boundaries between oneself and attractive others.

In the current investigation, we adapted an experimental paradigm used in a seminal study by Uddin, Kaplan, Molnar-Szakacs, Zaidel, and Iacoboni [10]. Participants were presented with digital morphs blending their own face with that of a gender-matched unfamiliar individual, displayed in a randomized order. They were instructed to press one button with their right hand if the image resembled their own face and another button if it resembled that of the unfamiliar person. Importantly and differently from Uddin and colleagues’ study [10], we morphed participants’ self faces with gender-matched unfamiliar faces that had undergone cosmetic enhancements, specifically dermal fillers treatments. Non-surgical cosmetic procedures, e.g. injectables such as botulinum toxin (Botox) and hyaluronic acid, laser skin treatments, and cryolipolysis (fat freezing) are indeed becoming more prevalent. Additionally, there is some indication of a rising trend in the number of young adults opting for cosmetic procedures in the last years [11]. For instance, facial beautification induced by plastic surgery, cosmetics or retouching can substantially alter the appearance of face images, thus impacting upon people’ ability to recognise themselves. Cosmetic treatments can significantly influence an individual’s psychological well-being. While those who opt for cosmetic interventions typically find satisfaction with the outcomes, experiencing psychological improvements, a subset of patients does not feel this way. Conditions like body dysmorphic disorder (BDD) have been recognized as predictors for unfavourable psychological outcomes, an increased likelihood of dissatisfaction with the results, and a propensity for repeated requests for surgical interventions [12, 13].

In this study, we wanted to explore whether self-enhancement, here defined as greater self-identification with positive physical attributes, in this case, a cosmetically enhanced face, could significantly alter self-other discrimination, in individuals keen to seek cosmetic procedures and with higher dysmorphic concerns. To this aim, we examined the relationship between self-face representation with individual differences in attitudes towards cosmetic surgery acceptance and body dysmorphic concerns. We also considered the potential impact of varying durations in viewing facial morphs—shorter versus longer times—on self-face recognition. This consideration stems from the idea that different perceptual and cognitive processes might be engaged during the self-recognition task depending on the duration of the viewing period. Therefore, in alignment with Feusner et al. [14], we systematically manipulated the viewing times (500 msec vs. 2000 msec) for each digital morph. This variation was grounded in the assumption that self-face recognition could be distinctively influenced by reflective, time-consuming cognitive processes (as observed in longer viewing times) or by more perceptual, reflexive factors (as observed in shorter viewing times). A similar time processing manipulation was also used by a study of Onden-Lim, Wu, and Grisham [15] which compared images containing a range of different face (and body) parts selected to represent typical concerns in a female BDD population with disgusting images of bodily products. In this study, a dot probe procedure was used to investigate the relationship between dysmorphic concerns and selective attention to faces, attractive, unattractive, and disgusting images in a female student population. Crucially, they also manipulated stimulus presentation time to allow insight into the role of automatic vs. more controlled processing. According to the authors, shorter presentations should invoke more automatic responding, whereas longer presentations should involve more elaborative thinking. They found that dysmorphic concerns were associated with attentional biases toward faces, attractive and possibly unattractive appearance-related images when visual stimuli were presented for long durations (1000 msec). On the other hand, dysmorphic concern was found not to be associated with attention to appearance related features when visual stimuli were presented for short durations (200 msec). These results are also consistent with research demonstrating that individuals displaying greater dysmorphic concerns selectively attend to attractive stimuli [16], and further emphasize the importance of considering time processing in these mechanisms.

Individual differences in more favourable attitudes towards cosmetic surgery were measured by the Acceptance of Cosmetic Surgery Scale (ACSS, [17]), whilst body dysmorphic concerns were measured by the Dysmorphic Concern Questionnaire (DCQ, [18]). Previous research using the ACSS suggests a link between negative body image and more favourable views on cosmetic surgery among college women [19]. Furthermore, previous studies using the DCQ to identify non-clinical populations (including university students) deemed to be high on a continuum of body image concerns reported that individuals displaying greater scores to the DCQ showed abnormalities in visual processing of faces (bodies, objects and scenes) as demonstrated by a detail-focused processing bias, which may be associated with maladaptive fixation on small features in their appearance [20–22]. Based on the above literature, we postulated that shorter ’reflexive’ viewing times would lead to heightened self-identification thresholds, necessitating a greater portion of the participant’s own face in the image for recognition, in comparison to longer ’reflective’ viewing times. In turn these effects would be associated to attitudes toward cosmetic surgery and levels of body dysmorphic concerns.

## Materials and methods

### Participants

Thirty female participants aged 18 to 24 years took part to the study. We tested only female participants, given the reported higher incidence of body image concerns amongst women as compared to men [23] and also because women report a greater likelihood of willingness to undergo various cosmetic procedures as compared to men [24, 25], see also [26] for a comprehensive systematic review. Participants were recruited internally through the Liverpool John Moores University (LJMU) research participation system for undergraduate Psychology students in exchange for SONA credit points and externally through poster advertisements on social media and through individuals known to the researchers. Inclusion criteria were: i) having normal or corrected to normal vision (with glasses/contact lenses) ii) being right-handed, iii) having no history of or any form of neurological and psychiatric disorders (including BDD and EDs). Participants were right-handed, (self)reported normal or corrected to normal vision and they were in good health, were free of psychotropic or vasoactive medication, with no current or history of psychiatric or neurological disease. Written informed consent was obtained by all participants. Ethical approval for the study was obtained from the LJMU Department of Psychology Research Ethics Committee. Data were collected between 14th February 2019 and 31st April 2019.

### Self-other facial morphing task

Self-face stimuli were individually tailored for each participant and consisted of a series of static grey scaled images constructed from pictures of the subjects’ own face and the face of six gender- and age-matched stranger. Each participant was photographed using the same digital camera (Panasonic TZ5 Lumix), looking directly at the camera, and holding a neutral expression. They were instructed to remove their glasses and to pull their hair back if it fell on their face. The images were then photoshopped, using Adobe Photoshop 7.0 (Adobe System Inc., CA, USA; http://www.adobe.com) to remove any facial piercings, skin blemishes and any noticeable marks on the face. Following, these images were each cropped to a dimension of 864×1080 pixels and subjected to a procedure devised to systematically morph participants image (*self*-condition) with six faces of women (unfamiliar to the participants) that had undergone non-invasive cosmetic lip and chick filler procedures (*other* condition). In this procedure, participant’s facial images were morphed in 20% increments up to 100%. Using the *FantaMorph* deluxe edition software (http://www.fantamorph.com), to create on whole image illusion, points on the participants’ faces, such as the corners of the mouth and jaw, were matched with identical points on the face using the face locator feature. As a result, a 20% morph with the other woman’s face produced a face that was 80% of the participant and 20% of the other woman. This morphing procedure produced 36 faces in total, 1 image morphed in stages of six (0%,20%,40%,60%,80%,100%) with six of the other women The other women photos were chosen making sure individuals with varying levels of lip and cheek fillers were used. The images were also selected to ensure that they could be gender- and age-matched to all participants. To achieve this, we initially selected seven emotionally neutral faces of women standing on a frontal pose who were chosen through social media and/or conducting an Internet search with the search terms “young woman face” “cosmetic enhancements”, “lip fillers” and “cheek fillers”. We excluded one out of seven faces because the image had poor resolution quality. We then collected attractiveness and cosmetic enhancement ratings (indicating the extent to which the observer believed the person in the picture had undergone cosmetic procedures, such as dermal fillers) of the facial stimuli from a separate group of 12 women, who were of comparable age to our experimental group (Mage = 19.58yrs, SD = 0.79). For our final set, we retained the images of 6 women, three of whom had undergone lip fillers and 3 who had undergone cheek fillers at variable levels. The mean ‘attractiveness’ ratings for the faces on a VAS scale of 0–100 was 60.58 (SD = 20.39). Whilst the mean ‘cosmetically enhanced’ ratings for the faces on a VAS scale of 0–100 was 52.89 (SD = 28.09). After the morphed images were produced, these were each further cropped on Adobe Photoshop 7.0 to 50×50mm, using the ellipse circle to produce an overall face, removing the background of the original image and a uniform grey background was created across all 36 images. All images were grey scaled to control for differences in skin tone. They were also mirror-reflected, in keeping with the ecological constraints of one’s visual experience for the own face, and the relative preference for mirrored views for self-face recognition [27].

### General procedure

**Fig 1:**
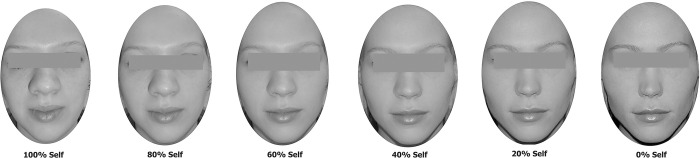
Example of a participant’s face (100% self and 0% other) morphed in six steps with an unknown, same-gender and cosmetically enhanced face (0% Self and 100% other) resulting in six degrees of morphing between the two faces (from left to right), with increasing % of other. Eyes are blurred in the images for privacy reasons.

The study consisted of two lab sessions. At day 1, participants’ picture was collected and processed for the stimuli preparation and at day 2, participants returned to the lab to complete the facial morphing task and the two self-reports. There was a maximum of two-weeks period between day 1 and day 2 for the completion of the study. Participants arrived at the first lab session and were asked to complete the participant information sheet and sign a consent form. Following this, participants filled out a demographic data questionnaire followed by measurements of their height and weight obtained from a calibrated digital scale (OMRON BF511) and a stadiometer, for Body Mass Index (BMI) calculation. When they returned for the second session, they were seated in a dimly lighted testing room in front of a 19-inch LCD monitor (resolution 1,027 × 768 pixels, screen refresh frequency at 60 Hz) at approx. 55cm away from the computer screen. The whole experiment was created in and controlled by E-Prime 2.0 Professional Software (Psychology Software Tools, Pittsburgh, PA). At the beginning of the experiment, they were given a standardized set of instructions and practiced with a set of 5 faces per each short (500 msec) and long (2000 msec) viewing times (total 10 trials). Each trial started with a fixation cross displayed for 500 msec, followed by a morphed face presented for 500 msec or 2000 msec depending on the experimental block, followed by a visual mask, which was presented for 500 msec until the response was given. Participants were told that they would be presented with a series of faces and would need to click as quickly as possible either the left-hand side of the mouse if the face was them (self) or would click the right-hand side of the mouse if they believed the face was the other woman (other). The response time was unlimited. The order of administration of the two blocks corresponding to the two viewing times (500 msec vs 2000 msec) was counterbalanced. Considering a 6 morphing levels × 6 models, 36 digital morphs, within each block (2 blocks) in a completely randomised fashion. Overall, across all conditions of viewing times and blocks, a total of 144 morphs was presented. Examples of morphed stimuli are reported in Fig 1.

At the end of the experiment, participants were asked if the ‘unfamiliar face’ was truly unfamiliar. All participants confirmed being unfamiliar with any of the 6 faces. Finally, participants completed the two self-report ACSS and DCQ scales. They were then thanked for their time and presented with a debrief sheet. All procedures required approximately 45 minutes to be completed.

### Self-report questionnaires

#### The Acceptance of Cosmetic Surgery Scale

The Acceptance of Cosmetic Surgery Scale (ACSS, [17]) consists of a total of 15 items which are rated on a 7-point Likert scale (1 = strongly disagree, 7 = strongly agree). Attitudes towards cosmetic surgery are measured by three subscales consisting of: 5 ‘Intrapersonal’ items, which measure attitudes related to the self-oriented benefits of cosmetic surgery (e.g. “If cosmetic surgery can make someone happier with the way they look, then they should try it’), 5 ‘Social’ items, that measure social motivations for having cosmetic surgery (e.g. “I would seriously consider having cosmetic surgery if my partner thought it was a good idea”) and 5 ‘Consider’ items, which assess the likelihood that an individual would consider having cosmetic surgery (e.g. “I have sometimes thought about having cosmetic surgery. Scores from each ACSS domain are averaged to compute a mean score for overall acceptance of aesthetic surgery (ACSS total). Previous work has shown that the ACSS has high internal consistency, good test–retest reliability after three weeks, and good convergent and discriminant validity [17]. The internal consistency for the current study was relatively high (ϑ = .92). We included this scale with the aim to determine whether women’ favourable attitudes toward cosmetic surgery were associated with greater likelihood to recognize an attractively enhanced version of their own face.

#### Dysmorphic Concern Questionnaire

The Dysmorphic Concern Questionnaire (DCQ, [18]) is a short, 7-item questionnaire used to measure an individual’s concern towards their physical appearance. The seven-items self-report questionnaire, in the form of a 4-point Likert scale rating from 0–3 (0 = not at all; 1 = like most people; 2 = more than other people; 3 = much more than other people) measures the degree to which the individual regards themself to be misshapen, concerns regarding bodily malfunctioning, the extent to which they have consulted with medical professionals, the degree of which they worry about their concerns and whether they spend time concealing their deficits. Total scores range from 0 to 28 with a critical value of 9 usually indicating clinical concern [28]. The DCQ has a relatively high internal consistency (ϑ = .82) [18]. This scale was included to determine whether women’ levels of dysmorphic concerns were associated with greater likelihood to recognize an attractively enhanced version of their own face.

### Data handling

For each morphing level, we recorded the percentage of ’self-other’ responses, indicating how frequently each morphed image was identified as ’self’ or ‘other’ For morphing levels 0, 20, and 40% “self” was considered the correct response, whereas for morphing levels 60, 80, 100% “other” was considered the correct response. Following this, a binary logistic regression was used to model the probability of "self-other" identification across morphing levels for both exposure times. The response variable represented whether the face stimuli were categorized as ’self’ or ’other’ by participants. For each participant, we then calculated the slope using a psychometric function fitted through maximum likelihood estimation for the Weibull distribution and we investigated transition points, Point of Subjective Equality (PSE), as well as the Just Noticeable Difference (JND) in self-other recognition across different morphing percentages for both viewing times. Finally, we ran Pearsons’s correlations (Bonferroni-corrected) and separate multiple regressions to explore whether ACSS total and DCQ scores were significant predictors of PSE for each viewing times. All analyses were conducted within the R programming environment (Version 4.0.3; R Core Team, 2018) using the afex package (Version 1.0–1; [29]). Figures were generated utilizing the ggplot2 package (Version 3.3.3; [30]).

## Results

### Demographic and self-report measures

The mean age for our sample was 21.1yrs (SD = 1.6), whilst the mean BMI was 23.92kg/m (SD = 4.34), which falls in the normal-weight category according to the to the World Health Organization’s BMI classification scheme. Regarding the scores obtained to the ACSS scale, the mean score for the Intrapersonal motives scale (perceived personal benefits offered by cosmetic surgery) was 5 (SD = 1.27), followed by a mean score of 4.62 (SD = 1.56) for the consideration subscale (perceived likelihood of applying for cosmetic surgery in the future) and a score of 3.68 (SD = 1.45) for the social motives subscale. Finally, the mean score for the ACSS-total was of 4.44 (SD = 1.25). The mean DCQ score was 10.33 (SD = 5.79, range: 0–20) which is slightly higher than the critical level of high dysmorphic concerns, with scores above 9 indicating clinical levels of body image concerns [18]. Finally, a strong, positive correlation was observed between DCQ and ACSS-total scores (*r* = .694, *p* < .004), so that the greater dysmorphic concerns, the more positive the acceptance towards cosmetic surgery.

### Self-recognition task performance

**Fig 2:**
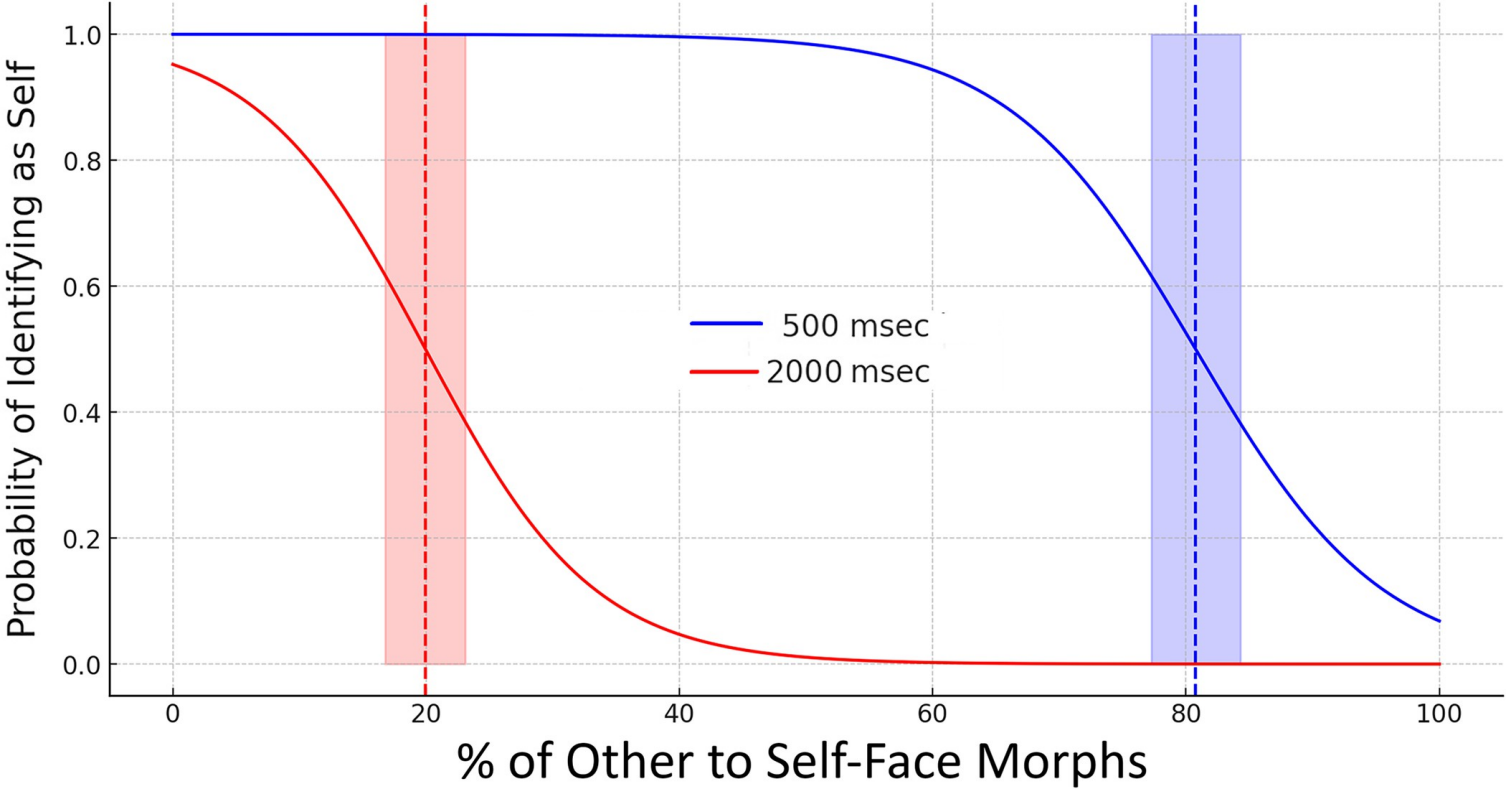
The % in the x-axis represents the % of other in the self-face. Blue and red solid lines represent the probability of ‘Self’ identification across the six morphing levels (%), depending on shorter (500 msec) vs longer (2000 msec) viewing times. Blue and red dashed vertical lines indicate the Point of Subjective Equality (PSE), for shorter (500 msec) vs longer (2000 msec) viewing times. Blue and red shading areas indicate the Just Noticeable Difference (JND) respectively, for shorter (500 msec) vs longer (2000 msec) viewing times.

A visual inspection of the data shows participants successfully completed the self-recognition task (see Fig 2). As anticipated, they identified digital morphs that were 100% morphed with another’s face as “other.” Notably, the frequency of “self” identifications diminished progressively with the increasing degree of morphing, notably, depending on the two viewing times.

### Logistic regression analysis

To characterize the distribution of self-other responses across the two viewing times, we calculated the morph level at which 50% self-response was recorded. A paired sample t-test revealed that the morph level at which self-face response reached 50% for 500 msec viewing time was significantly higher compared to the morph level at which 50% self-face response for 2000 msec viewing time was reached (t  =  14.98, p < 0.001).

A logistic regression analysis was conducted to examine the effect of morphing percentage and viewing times on participants’ self-recognition. The model included morphing levels, viewing times, and their interaction as predictors. Results indicated that both morphing levels (B = 4.64, SE = 0.85, z = 5.47, p < .001) and viewing times (B = 3.99, SE = 1.06, z = 3.76, p < .001) were significant predictors of self-other recognition. The interaction between morphing levels and viewing times was not statistically significant (B = 0.5, SE = 1.06, z = 3.76, p = .707). These results suggest that both morphing percentage and exposure time independently influence the participants’ ability to recognise the face, with no significant interaction effect observed.

Although the interaction did not significantly predict PSEs for the two viewing times, the notable significant difference at the morph level where self-face recognition reached 50% between the two viewing times provides a strong basis for a detailed examination. Consequently, we opted to analyse the effects of morphing percentage on the response separately for each viewing duration. This method facilitates a more precise understanding of how morphing levels impact discrimination performance independently at each viewing time, thereby offering deeper insights into the dynamics of facial self-other discrimination under various temporal conditions. For the 500 msec exposure duration, the morphing levels significantly predicted the likelihood of ’self-other’ recognition, B = 0.136, SE = 0.025, z = 5.47, p < .001. The intercept was also significant, B = -10.97, SE = 2.01, z = -5.45, p < .001, indicating the log-odds of ’self-other’ recognition at 0% morphing. Similarly, for the 2000 msec viewing time, the morphing levels was a significant predictor, B = 0.15, SE = 0.029, z = 5.23, p < .001. The intercept for this duration was also significant, B = -2.99, SE = 0.66, z = -4.53, p < .001, reflecting the baseline log-odds of recognizing the face as ’self-other’ at 0% morphing.

These findings elucidate how the temporal dynamics of viewing times influence self-face recognition, with longer viewing times requiring less morphing for participants to perceive the face as ’other.’

### Transition points and Point of Subjective Equality (PSE)

The analyses of transition points in self-other recognition across different morphing percentages for both viewing times revealed that for the 500 msec viewing time, the PSE was at approximately 80.76% morphing, indicating an even likelihood of ’self’ or ’other’ recognition. For the 2000 msec viewing time, a much lower PSE of around 19.95% was observed, suggesting a swifter shift to ’other’ recognition. The JND for each exposure time is as follows: for the 500 msec exposure time, the JND is approximately 7.37%. This value indicates the smallest change in morphing percentage that leads to a noticeable change in the participants’ response. For the 2000 msec exposure time, the JND is approximately 6.67%, suggesting a slightly lower sensitivity to changes in morphing percentage compared to the 500 msec exposure time. Moreover, the slope in a logistic regression context and in our case represents the change in the log odds of identifying the face as “self” or “other” for a one-unit change in the morphing percentage. The analysis of sigmoid curve slopes at the PSE for both durations showed that for 500 msec viewing time the slope was ≈ 0.136, whilst for the 2000 msec viewing time the slope was ≈ 0.15. Since from the logistic regression analysis, the coefficient for the interaction term was not statistically significant (*p* = .707), the difference in slopes (the effect of morphing levels on the likelihood of recognizing the face as ’self-other’) between the two viewing times is not significant (see Fig 2).

In summary, our findings illustrate a significant interplay between viewing times and self-other recognition within the context of (cosmetically enhanced) digital morphs. Shorter viewing durations of 500 msec which requires more reflexive, immediate self-recognition, resulted in a diminished perceptual discrimination capability, necessitating a higher threshold of ’other’ morph percentage for individuals to differentiate self from other. Conversely, when reflective, complex decision-making process are involved (2000 msec), participants’ discriminatory accuracy was enhanced, allowing for a lower ’other’ morph percentage to suffice for self-recognition. This dichotomy underscores the critical role of viewing times in modulating reflective vs. reflexive perceptual processes underlying identity recognition, highlighting a nuanced dynamic where longer exposure times facilitate more refined and sensitive distinctions between self and other representations.

### Correlations and multiple regression analyses

Pearson correlation coefficients revealed significant relationships between ACSS total scores, DCQ scores and individual PSEs for the two shorter (500 msec) and longer (2000 msec) viewing times. Specifically, ACSS total scores showed a positive correlation with the 500 msec PSE (r = 0.554, p < .005) and a negative one with the 2000 msec PSE (r = -0.386, p = .035). DCQ scores positively correlated with the 500 msec PSE (r = .588, p < .001) and negatively with the 2000 msec PSE (r = -.365, p = .047). To correct for multiple comparisons, a Bonferroni correction was applied, setting the alpha level at 0.025. Even after this adjustment, the correlations remained statistically significant, indicating a reliable relationship between PSE for 500 ms and 2000 ms, and both DCQ scores and ACSS total scores. These correlations suggest that perceptions in the self-recognition task vary with dysmorphic concerns and attitudes towards cosmetic surgery, crucially depending on viewing times.

**Fig 3:**
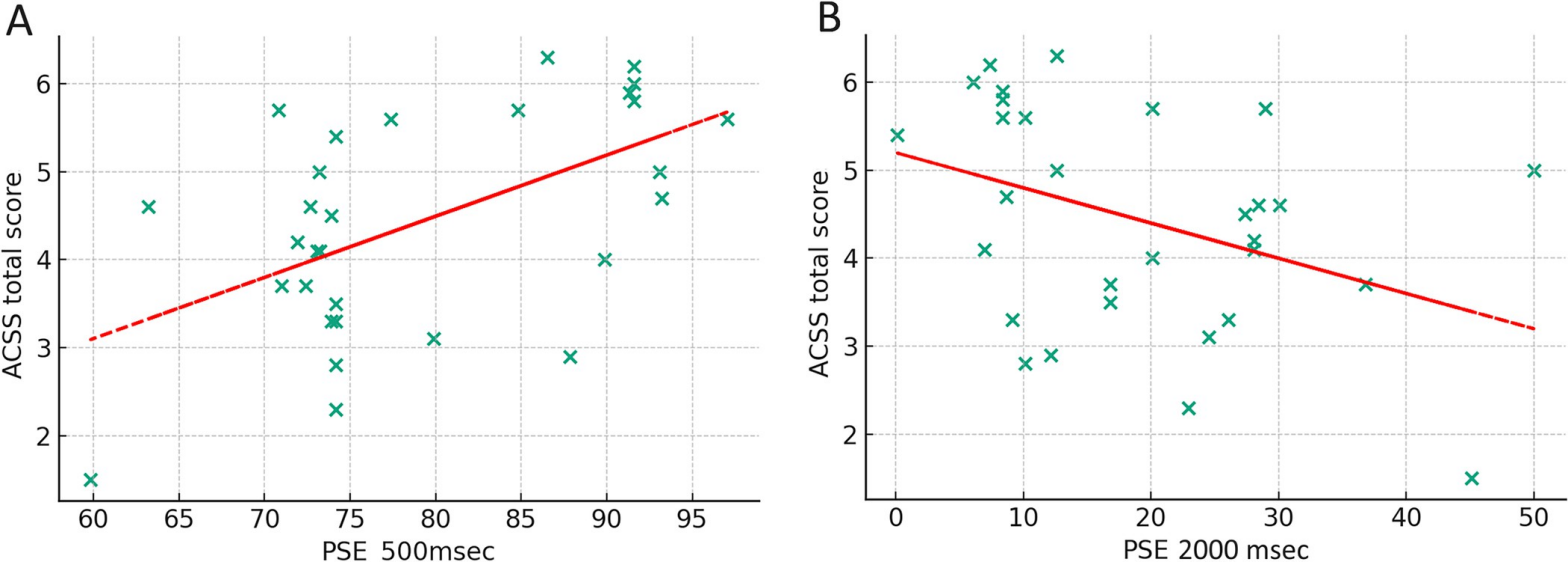
Correlation between the Point of Subjective Equality (PSE) and scores obtained at the Acceptance of Cosmetic Surgery Scale (ACSS), for A) shorter viewing times (500 msec) and B) for longer viewing times (2000 msec).

Linear regression models provided further insights. The linear regression model examining the relationship between the 500 msec PSE and ACSS total scores accounted for 29.6% of the variance (*R^2^* = .296, *F*(1, 29) = 11.76, *p* = .002). This indicated a moderate correlation between the 500 msec PSE and ACSS total scores. The positive slope suggested that higher ACSS scores are associated with higher PSE values (greater levels of information for the other morph condition is needed) at the 500 msec viewing time. The ACSS total scores and 2000 msec PSE model was also significant, accounting for 14.9% of the variance (R^2^ = .149, F(1, 29) = 4.9, p = .035), suggesting a notable influence of attitudes towards cosmetic surgery on recognition at longer exposure times, with more positive attitudes towards acceptance of cosmetic surgery correlating with ’other’ identification at a lower morphing percentage (see Fig 3).

**Fig 4:**
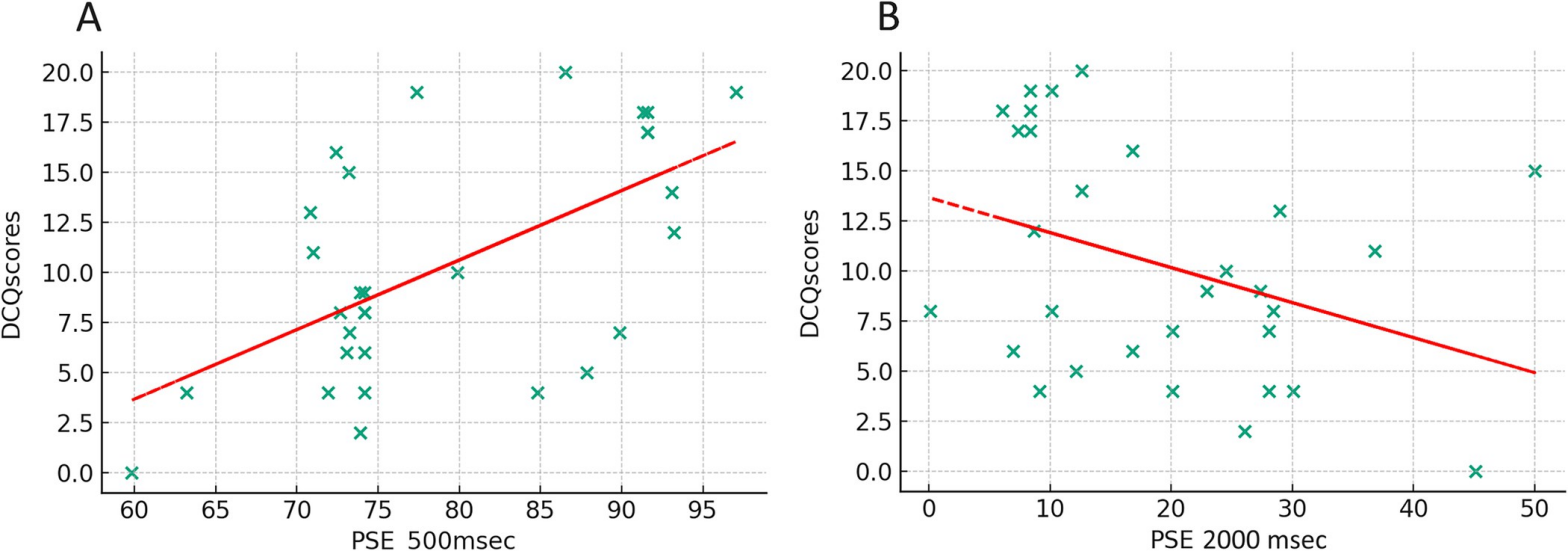
Correlation between the Point of Subjective Equality (PSE) and scores obtained at the Dysmorphic Concerns Questionnaire (DCQ), for A) shorter viewing times (500 msec) and B) for longer viewing times (2000 msec).

The model for the 500 msec PSE and DCQ scores accounted for 34.6% of the variance (*R^2^* = .346, *F*(1, 29) = 14.82, *p* < .001). This suggested a moderate influence of body dysmorphic concerns on self-recognition at shorter viewing times, with higher dysmorphic concerns correlating with higher PSE (greater levels of information for the other morph condition is needed). For the 2000 msec PSE and DCQ scores, 13.3% of the variance was explained (R^2^ = .133, F(1, 29) = 4.3, p = .047), indicating a slightly lesser but significant influence of dysmorphic concerns on recognition at longer viewing times, with higher body dysmorphic concerns correlating with lower PSE (see Fig 4).

Taken together these findings indicate a substantial influence of both attitudes toward cosmetic surgery and body dysmorphic concerns on how individuals perceive images of themselves post-cosmetic enhancement, with the effects varying across different viewing times. These associations underscore the intricate interplay between psychological factors and self-perception, particularly within the realm of negative body image and attitudes toward cosmetic surgery.

## Discussion

In this study, we investigated the complex interplay between self-face recognition, attitudes towards cosmetic surgery, and concerns over body dysmorphia. We employed a methodology that utilized morphed self-other images of individuals who underwent cosmetic enhancements and presented digital morphs at shorter and longer viewing times. Specifically, self-face representation was measured as the slope for self-recognition, varying as a function of available physical self-related information. In turn, this was manipulated by creating degrees of morphs with differing percentages of self-related information against that of women who underwent cosmetic enhancements (i.e., dermal fillers). Furthermore, the inclusion of two different viewing times of digital morphs allowed to test ‘reflective’ vs ‘reflexive’ processess of self-face processing, respectively at longer (2000 msec) and shorter (500 msec) viewing durations. In doing so, we took advantage of the steepness of the slope, and of the subjective perceptual threshold ‘PSE’, calculated from the self-recognition responses across the different degrees of morphs, which provided a measure of stimulus range over which the participant shifts between self and other boundaries depenging on the two viewing times. Accordingly, a steeper slope indicated reduced overlap between self and other, with greater PSE values indicating greater difficulties in recogniting self vs. other digital morphs. We finally looked at the slope in relation to individual differences in attitudes towards cosmetic surgery and body dismorphic concerns.

We found that when digital morphs are presented for shorter viewing periods of 500 msec, during which a more perceptually driven, reflexive type of self-recognition processing is required, individuals displayed a higher perceptual threshold of ’other’ morph percentage to correctly differentiate between self and other. In fact, the perceptual threshold corresponding to a 50% positive response rate was 80.76%, that is participants had more difficulties to detect facial changes and required images that contained more “other” to recognize themselves. These results are in keeping with Epley and Whitchurch [5]’ study which showed that when photographs of participant’s faces are morphed with attractive faces, they were more likely to identify the attractive morphs as the own face compared to their actual face. This is also consistent with previous studies using similar self–other facial morphing tasks which reported an egocentric bias, according to which facial morphs are judged to look more like “self” when morphed with a more attractive relative to a less attractive face [5, 6].

On the other hand, we found that when reflective, complex decision-making process are in play (viewing times of 2000 msec), participants’ face discrimination ability is enhanced, allowing for a lower ’other’ morph percentage to suffice for self-recognition. For this condition, only 19.95% of the other face in the morph was sufficient for the participants to start shifting their judgements. These results may imply that during longer viewing times, participants may engage more in a meticulous analysis of the observed facial stimulus. Accordingly, this fine-grained analysis may enable the computation of the overall distance between the observed face and the internal self-face representations, facilitating the generation of a precise and conclusive response. Taken together, these results may speak in favour of the hypothesis that a different engagement of local- vs. global-based processing is required to resolve self- to other-face discrimination, if one takes into account the two short and longer viewing times. For instance, several investigations using a face inversion task, according to which inverting a face can severely disrupt holistic, configural-based processing for faces [31, 32], and consequently causing poor recognition, have shown that the processing of the own face relies on featural information [33, 34]. Brédart [35] shows that while we rely mainly on configuration in recognizing others, local information is also important in recognizing ourselves. Furthermore, evidence from event-related potential studies reported an increased N170 amplitude for the own face which is also consistent with the idea that the own face is processed in a more featural manner (e.g., [36–38]). A study by Beilharz and colleagues [22] further demonstrated that increases in body image concerns, as defined by DCQ score, was linked to reduced face (and body) inversion effects, as demonstrated by superior accuracy rates for inverted stimuli. Additionally, an unexpected association was revealed between DCQ scores and reaction time in the case of upright body stimuli in the discrimination task, indicating that as individuals increased in body image concerns, so did the amount of time spent processing the upright body images. The authors suggest that this longer looking duration for upright bodies might be related to the high dysmorphic concerns tendency of comparing one’s appearance to those of other people, as these individuals often spend a debilitating amount of time examining their own appearance in this typical orientation [16], an association that requires further exploration. Although we cannot draw definitive conclusions here, we speculate that the dichotomy between configural and local-based self-recognition processes underscores the critical role of exposure duration in modulating reflective versus reflexive perceptual processes underlying identity recognition. This highlights a nuanced dynamic where longer viewing times facilitate more refined and sensitive distinctions between self and other representations.

Interestingly, our results also indicate that both positive attitudes towards cosmetic treatments and body dysmorphic concerns significantly impacted individuals’ perceptions of morphed images of themselves, with influences varying based on viewing times. During ’reflexive’ viewing times (500 msec), we observed positive associations with both ACSS and DCQ scores. This suggests that individuals with more heightened pursuit of cosmetic treatments and greater body dysmorphic concerns demonstrate higher PSE values, that is greater levels of ‘other’ evidence information is needed to recognise themselves. At longer ’reflective’ viewing times (2000 msec), we noted a contrasting, opposite trend, where higher PSE values were predicted by lower desire to pursue cosmetic surgery and fewer dysmorphic concerns.

The findings on the relationship between self-face recognition and attitudes towards cosmetic treatments resonate with existing research which support a link between acceptance of cosmetic surgery and personality and individual difference predictors. For instance, Cash, Goldenberg-Bivens, and Grasso [19] had previously identified a correlation between body image perceptions and attitudinal dispositions towards cosmetic surgery, focusing primarily on the psychological dimensions of self-esteem, conformity, and self-assessed attractiveness [19]. These results are also consistent with past research, demonstrating that self-recognition skills could be disrupted in individuals suffering from AN [8, 39, 40] and from BDD [14]. For example, a recent study by Hirot and colleagues [8] reported that patients with AN had more difficulties to detect facial changes and required images that contained more “self” to recognize themselves, a deficit that positevely correlates with their nutritional state. Furthermore, a more relevant study by Feusner and colleagues [14] reported that individuals with BDD exhibit an asymmetry between detail and global processing of their face. This imbalance was particularly evident during prolonged face presentations, where there is ample time for the encoding of intricate details. In contrast, with brief stimuli presentations, the limited time available may restrict detailed processing, allowing only for holistic processing. The fact that the inversion effect remains normal in BDD subjects during short viewing durations implies that the detail vs. holistic processing imbalance may be a dynamic occurrence, manifesting primarily in situations involving extended viewing periods. Clinically, this dynamic seems to be prevalent in the daily routines of individuals with BDD, who often dedicate substantial time, ranging from minutes to hours, scrutinizing themselves in mirrors and reflective surfaces [41].

Our study adds a new layer to this understanding by linking these attitudes directly to the mechanisms of self-face recognition. This may suggests that the perception of one’s physical self is not only a reflection of internal self-concept but also deeply influenced by external societal and cultural norms regarding beauty and aesthetics. The relationship with body dysmorphic concerns, while subtler, points to the intricate ways in which body image disturbances intersect with self-perception and is consistent with literature that examines the psychological repercussions of body image issues, particularly in the context of cosmetic treatments [18, 42, 43].

Although this study was the first to assess self-recognition of cosmetically enhanced faces in relation to individual differences of acceptance toward cosmetic treatments and body dysmorphic concerns, limitations have been identified. First, one limitation to our study pertains to the response mapping (‘self’ with left-side key and ‘other’ with right-side key) which was not inverted. Previous literature reported that self-related material is processed in the right hemisphere, which makes subjects faster in self-recognition when using their left hand [44, 45]. Because each hand is predominantly controlled by the motor cortex in the contralateral cerebral hemisphere, the finding suggests that the right hemisphere may dominate self-face recognition. Here, it should be noted that participants used their dominant hand (right-hand) to discriminate whether the digital morphs were of their face or someone else. Whilst we did not compute measures of reactions times, we cannot exclude that this might have had an effect on our results. Research has shown that when hand differences are not controlled for, self-faces remain robust (i.e., exhibit shorter reaction times) compared to strangers’ faces, even during repeated presentations [46].

Moreover, in addition to the explanations discussed regarding reflective versus reflexive and global versus local processing, it is crucial to consider task difficulty as a potential confound. The two differing viewing times may have influenced participants’ response criteria, shifting from a liberal to a conservative approach in discriminating the self from other as morph levels change. This shift might explain the observed pattern of results and represent a more general, and possibly pivotal, aspect of our findings. Acknowledging this possibility enhances our understanding of the factors influencing facial self-other discrimination and underscores the need for further investigation into how temporal dynamics affect response strategies.

Furthermore, it should be noted that even SNARC-like effects could have affected our results. In fact, valence (other than the magnitude) associated with a stimulus appears to be spatially connoted so that relatively negative concepts are associated with the left side of space, and relatively positive concepts are associated with the right side [47, 48], at least among right-handed individuals [49]. Accordingly, one may speculate that cosmetically enhanced (positive) self and other (negative) faces would be more easily associated with the left and right side of space, respectively [50]. On the other hand, a more recent study by Dalmaso and Vicovaro [51] reported that processing of face age failed to be translated into a clear left-to-right horizontal spatial representation. Given that faces are complex stimuli whose social interpretation is multifaceted, further understanding of the horizontal representation of attractive/positive ‘self’ could be a valuable avenue for future research.

The study’s focus on a specific demographic group (young, female participants) limits its generalisability to male populations. Previous research has indeed suggested that women show higher interest in cosmetic surgery and are more likely to report having had a procedure [24, 25, 52]. Data from the American Society for Aesthetic Plastic Surgery [53] show an overwhelming majority of cosmetic surgery patients are female. Regarding non-surgical cosmetic procedures, these are also more popular for women. These statistics motivated the present research to focus on female participants only. However, very little research in cosmetic procedures has included men. Therefore, it is not clear how men relate to or consider altering their appearance for aesthetic reasons and future studies should address self-face recognition abilities in men. Future research should also aim to include a broader and more diverse range of participants to explore the universality of these findings. This would provide a more comprehensive understanding of the impact of societal beauty standards across different cultures, genders, and age groups. While the study precisely controlled for an array of variables influencing self-recognition, it is worth acknowledging that additional factors, such as body dissatisfaction, self-esteem, and perceived self-attractiveness, may also exert an influence.

It should be noted that our study is likely not adequately powered for correlational analyses given the relatively small sample size of participants. However, we do provide some preliminary correlational evidence for strong associations between DCQ and ACSS scores and self-other visual discrimination performance. Nevertheless, such evidence should be cautiously interpreted.

Notably, it is worth mentioning that the implications of our findings extend beyond the realm of individual psychology, touching on broader societal and cultural issues. The growing prevalence of cosmetic treatments and the pervasive influence of media on beauty standards highlight the need for a critical examination of how societal norms shape individual self-perception. This is particularly relevant for vulnerable populations, such as young women, who are often most influenced by these trends and from emerging phenomenon, including the so called ‘Snapchat Dysmorphia’ which raises a concern about the negative effects of social media applications, such as Snapchat and Instagram, on users related to the choice of plastic surgeries [54]. This is especially important given that procedures like these come with inherent medical risks, such as infections, and the potential for adverse psychological outcomes, like disappointment [55]. Additionally, there are psychosocial consequences, including negative perceptions from others and stigmatisation [56].

To conclude, our research offers critical insights into the psychological processes underpinning self-perception, particularly in the context of cosmetic enhancements and concerns over body dysmorphia. The study elucidates the complex dynamics between self-image, societal influences, and psychological well-being, contributing significantly to the existing body of literature. These findings have important implications for both clinical practice and societal discourse, underscoring the need for greater awareness and understanding of the psychological impact of beauty standards and cosmetic surgery in modern society.
